# NKL Homeobox Gene VENTX Is Part of a Regulatory Network in Human Conventional Dendritic Cells

**DOI:** 10.3390/ijms22115902

**Published:** 2021-05-31

**Authors:** Stefan Nagel, Claudia Pommerenke, Corinna Meyer, Hans G. Drexler

**Affiliations:** Department of Human and Animal Cell Lines, Leibniz-Institute DSMZ—German Collection of Microorganisms and Cell Cultures, 38124 Braunschweig, Germany; cpo14@dsmz.de (C.P.); cme@dsmz.de (C.M.); Hans.Drexler101@t-online.de (H.G.D.)

**Keywords:** AML, cell lines, homeobox, NKL-code, T-ALL

## Abstract

Recently, we documented a hematopoietic NKL-code mapping physiological expression patterns of NKL homeobox genes in human myelopoiesis including monocytes and their derived dendritic cells (DCs). Here, we enlarge this map to include normal NKL homeobox gene expressions in progenitor-derived DCs. Analysis of public gene expression profiling and RNA-seq datasets containing plasmacytoid and conventional dendritic cells (pDC and cDC) demonstrated HHEX activity in both entities while cDCs additionally expressed VENTX. The consequent aim of our study was to examine regulation and function of VENTX in DCs. We compared profiling data of VENTX-positive cDC and monocytes with VENTX-negative pDC and common myeloid progenitor entities and revealed several differentially expressed genes encoding transcription factors and pathway components, representing potential VENTX regulators. Screening of RNA-seq data for 100 leukemia/lymphoma cell lines identified prominent VENTX expression in an acute myelomonocytic leukemia cell line, MUTZ-3 containing inv(3)(q21q26) and t(12;22)(p13;q11) and representing a model for DC differentiation studies. Furthermore, extended gene analyses indicated that MUTZ-3 is associated with the subtype cDC2. In addition to analysis of public chromatin immune-precipitation data, subsequent knockdown experiments and modulations of signaling pathways in MUTZ-3 and control cell lines confirmed identified candidate transcription factors CEBPB, ETV6, EVI1, GATA2, IRF2, MN1, SPIB, and SPI1 and the CSF-, NOTCH-, and TNFa-pathways as VENTX regulators. Live-cell imaging analyses of MUTZ-3 cells treated for VENTX knockdown excluded impacts on apoptosis or induced alteration of differentiation-associated cell morphology. In contrast, target gene analysis performed by expression profiling of knockdown-treated MUTZ-3 cells revealed VENTX-mediated activation of several cDC-specific genes including CSFR1, EGR2, and MIR10A and inhibition of pDC-specific genes like RUNX2. Taken together, we added NKL homeobox gene activities for progenitor-derived DCs to the NKL-code, showing that VENTX is expressed in cDCs but not in pDCs and forms part of a cDC-specific gene regulatory network operating in DC differentiation and function.

## 1. Introduction

Homeobox genes encode transcription factors (TFs) which regulate basic processes of cell, tissue, and organ differentiation in the embryo and in the adult. They share the conserved 180 bp homeobox which encodes the homeodomain at the protein level mediating specific contacts to DNA and cofactors [1]. Comparison of homeobox sequences has been used to organize these genes into classes and subclasses. Accordingly, humans contain 48 NKL homeobox subclass genes which belong to the ANTP class [2]. The ANTP class represents the largest group of homeobox genes and additionally contains the clustered HOX genes, belonging to the HOXL subclass [2]. NKL factors share particular amino acids at certain positions in their homeodomain. Furthermore, they possess an N-terminal motif which performs interactions with repressive cofactors of the groucho-family [1,2]. Specific expression patterns of homeobox gene subgroups have been reported for particular embryonic body parts. Indeed, HOX genes are expressed in the developing brain in an anterio-posterio pattern generating the HOX-code while DLX genes are expressed in the pharyngeal region in a dorso-ventral pattern generating the DLX-code [3,4].

We analyzed the physiological expression of NKL homeobox genes in hematopoiesis including stem cells, lymphopoiesis, and myelopoiesis and termed the pattern NKL-code [5,6,7]. This code comprises eleven genes and enables differentiation of normal and aberrant expression of NKL homeobox genes in leukemia and lymphoma patient samples. Recently, the basic roles of NKL homeobox genes in regulation of normal and abnormal developmental hematopoietic processes have been reviewed [8]. For example, NKL homeobox gene NANOG is normally expressed only in early stages of hematopoiesis and aberrantly activated in acute myeloid leukemia (AML) patients [7]. This gene operates in embryonic stem cells and is activated in generating induced pluripotent stem cells [9]. The micro-RNA gene cluster MIR17HG represents an activated target in neural stem cells and hematopietic cells [7,10]. HMX2 and HMX3 are no members of the NKL-code and ectopically expressed in subsets of AML [11]. Normally, these embryonic factors regulate the development of the inner ear [12,13]. In AML cells, they inhibit the expression of the myeloid differentiation gene EPX and perform oncogenic activation of the ERK-pathway [11]. HHEX and HLX represent the most prevalent NKL-code members, regulating major steps in both, myelopoiesis and lymphopoiesis [14,15,16]. Their deregulation has been reported for T-cell leukemia and Hodgkin lymphoma, respectively [5,17]. STAT3 operates as major activator of HLX expression. This impact plays an oncogenic role in anaplastic large cell lymphoma where STAT3 represents a hallmark factor [18].

Monocytes are generated in the course of myelopoiesis via common myeloid progenitors (CMP) and macrophage dendritic cell progenitors (MDP) and express the NKL homeobox genes DLX2, HHEX, HLX, NKX3-1, and VENTX [7]. They are able to differentiate into macrophages and dendritic cells (DC). These monocyte-derived dendritic cells (moDC) differ from progenitor-derived DCs in gene expression and function and are generated at sites of inflammation or ex vivo [19,20,21]. MDP-derived common dendritic cell progenitors (CDP) generate plasmacytoid dendritic cells (pDC) and conventional dendritic cells (cDC) via specific progenitors. The cDC-type is further divided in cDC1 and cDC2 subtypes, the latter representing the major population of cDCs in blood, tissues, and lymphoid organs [21]. The factors and processes regulating DC differentiation are mainly controlled at the transcriptional level [19]. Accordingly, several pDC- and cDC-specific transcription factors (TF) have been reported, including MYB, SPIB, and TCF4 as main regulators of pDCs while CEBPB, IRF2, and SPI1 (formerly PU.1) dictate the differentiation of cDCs [19,20,21,22].

Hematopoietic malignancies emerge from particular progenitor cells, executing deregulated proliferation and differentiation arrest while retaining many aspects of their progenitors. Thus, blastic plasmacytoid dendritic cell neoplasm (BPDCN) is derived from pDC-progenitors. This malignancy is rare and confers a poor prognosis [23]. In contrast, the existence of cDC-derived malignancies is unclear. However, gene expression analyses of Langerhans histiocytosis cells (LHC) indicated their origin from cDCs and experimental transformation of cDCs recapitulated phenotypes of LHC in mice [24,25,26]. This unsolved etiology may be related to the rarity of this tumor type and/or the lack of suitable markers.

Here, we analyzed expression data from primary hematopoietic samples and completed the NKL-code for progenitor-derived DCs, including pDCs and cDCs. Detailed examinations of VENTX expression and function showed that this NKL homeobox gene is part of a cDC-specific network.

## 2. Results

### 2.1. Expression Analysis of NKL Homeobox Genes in Dendritic Cells

**Figure 1:**
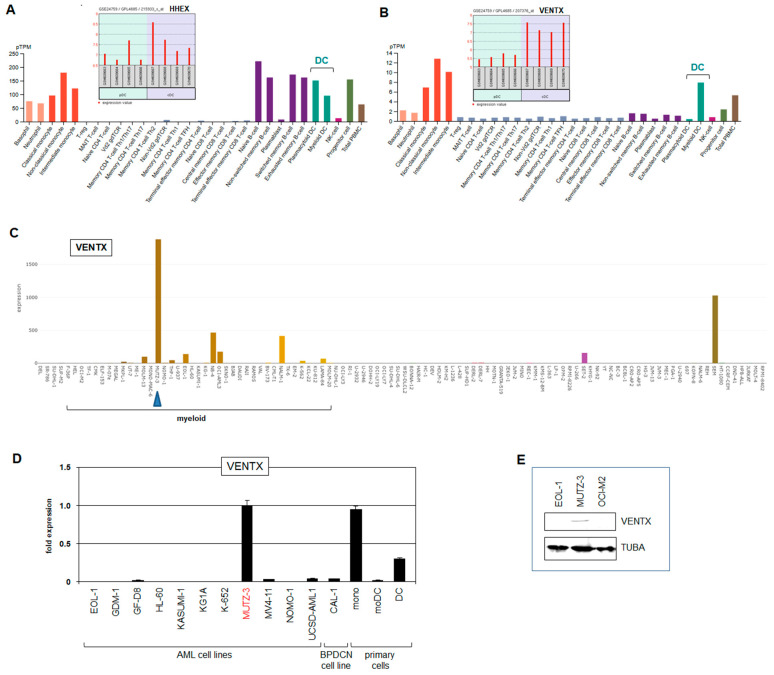
VENTX and HHEX expression. Expression analyses of NKL homeobox genes HHEX (**A**) and VENTX (**B**) were performed using public datasets from GEO (GSE24759) and the Human Protein Atlas. The data show that HHEX is active in both pDC and cDC entities while VENTX is expressed in cDCs but not pDCs. (**C**) VENTX expression analysis was performed using RNA-seq dataset LL-100. Conspicuous VENTX levels are shown for AML cell line MUTZ-3 (blue arrow head). The myeloid cell lines are indicated. (**D**) RQ-PCR analysis of VENTX in selected AML and BPDCN cell lines and primary cells including monocytes (mono), monocyte-derived DCs (moDC), and DCs. The indicated fold expression levels are relative to MUTZ-3 which was set to unity. (**E**) Western blot analysis of VENTX and alpha-Tubulin (TUBA) which served as loading control. VENTX protein was detected in MUTZ-3 while two control cell lines tested negative.

**Figure 2:**
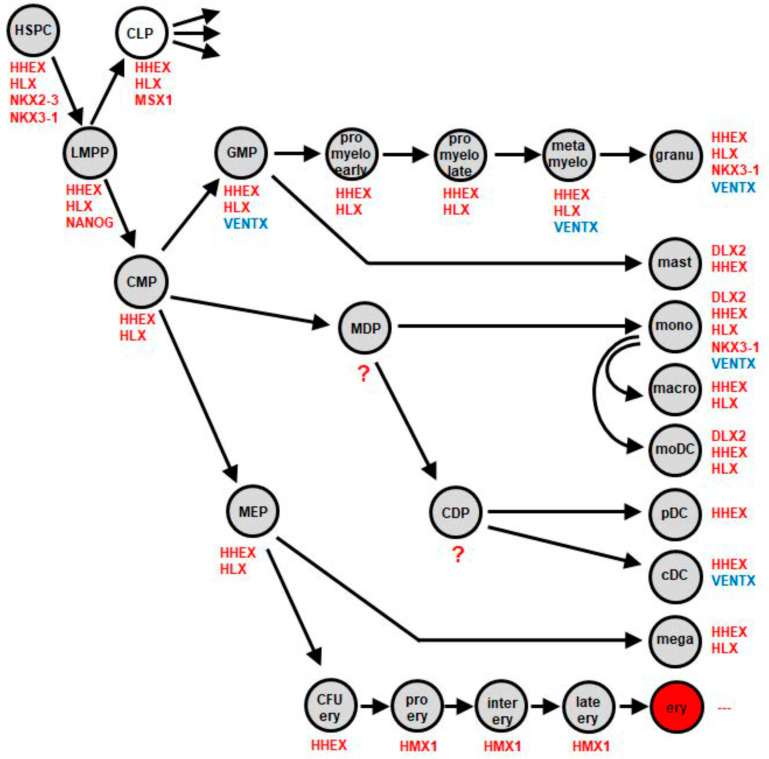
Myeloid NKL-code containing progenitor-derived DCs. This scheme shows the updated myeloid NKL-code and summarizes published data [7] with results from this study for progenitor-derived DCs including pDCs and cDCs. NKL homeobox gene VENTX is indicated in blue. cDC: conventional dendritic cell, CDP: common dendritic cell progenitor, CFU ery: colony forming unit for erythrocytes, CLP: common lymphoid progenitor, CMP: common myeloid progenitor, ery: erythrocyte, GMP: granulocyte and mast cell progenitor, granu: granulocytes, HSPC: hematopoietic stem and progenitor cell, LMPP: lymphoid and myeloid primed progenitor, macro: macrophage, MDP: monocyte and dendritic cell progenitor, mega: megakaryocyte, MEP: megakaryocyte and erythrocyte progenitor, moDC: monocyte-derived dendritic cell, mono: monocyte, pDC: plasmacytoid dendritic cell.

Recently, we reported the normal activities of NKL homeobox genes in myelopoiesis to extend the NKL-code for this hematopoietic lineage [7]. This analysis included moDCs but lacked pDCs and cDCs which derive from CDPs. To fill this gap, we analyzed NKL homeobox gene activities using public gene expression profiling dataset GSE24759 which contains data for pDCs and cDCs in addition to CMPs and monocytes. In both pDCs and cDCs, we detected active NKL homeobox gene HHEX while cDCs also expressed VENTX which was silent in pDCs (Figure 1A,B). RNA-seq based gene expression data from the Human Protein Atlas supported these findings for pDCs and cDCs, showing additional activities of both HHEX and VENTX in granulocytes and monocytes (Figure 1A,B). Thus, pDCs and cDCs differ in VENTX expression. These results were integrated into our scheme of the NKL-code for myelopoiesis (Figure 2), showing VENTX activity in cDCs, granulocyte macrophage progenitor (GMP), and mature granulocytes and monocytes [7]. Thus, moDCs and pDCs silenced VENTX, indicating that this NKL homeobox gene may play a suppressive role in the generation of these types of DC. In the following, we examined regulation and function of VENTX in cDCs.

### 2.2. Screening for Candidate VENTX Regulators

**Figure 3:**
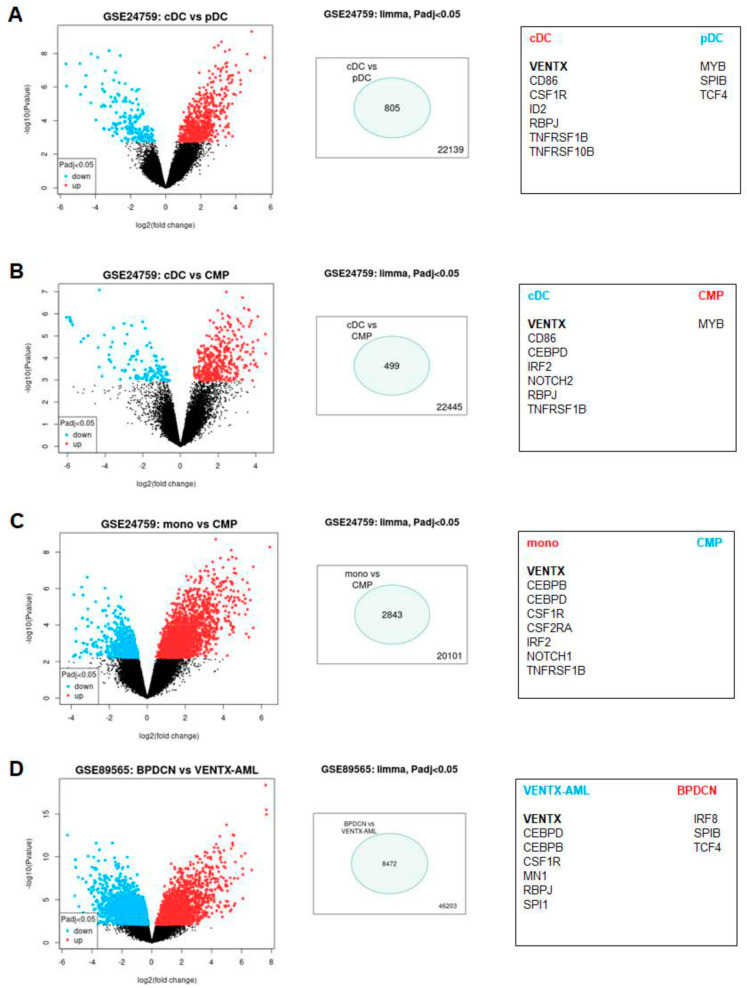
Identification of potential regulators of VENTX. Dataset GSE24759 was analyzed to identify significant differentially expressed genes between VENTX-positive and VENTX-negative hematopoietic entities. We used the online tool GEOR and compared (**A**) cDC versus pDC, (**B**) cDC versus common myeloid progenitors (CMP), and (**C**) monocytes versus CMP. (**D**) Dataset GSE89565 was used for comparison of six VENTX-positive AML versus 12 BPDCN patient samples. The results are presented as volcano-blot on the left, showing significant genes in blue and red. The numbers of significantly differently expressed genes are indicated in the middle. Selected gene candidates for each analyzed entity are listed on the right. VENTX is highlighted in bold letters.

To identify regulators of VENTX transcription, we analyzed expression profiling dataset GSE24759 using the associated online tool GEOR. We compared VENTX-positive versus VENTX-negative entities, namely cDC versus pDC, cDC versus CMP, and monocytes versus CMP (Supplementary Tables S1–S3, Figure 3A–C). Shortlisted genes are indicated for each comparison, demonstrating several similarities. Accordingly, VENTX expression correlated with CEBP, ID2, IRF2, NOTCH-signaling, TNF/NFkB-signaling, and CSF-signaling activities while the TFs MYB, SPIB, and TCF4 were suppressed in VENTX-positive samples (Figure 3A–C). Therefore, this approach highlighted these factors and pathways as candidate regulators of VENTX.

BPDCN is a myeloid malignancy derived from pDC [23]. Dataset GSE89565 contains gene expression profiling data from both BPDCN and AML patients. We analyzed these entities by GEOR, showing that VENTX was aberrantly expressed in 6/65 AML patients, while in all 12 BPDCN patients, VENTX was silenced. This finding corresponded to the absence of VENTX activity in normal pDC cells. Additional significantly expressed genes in the groups of VENTX-positive AML patients versus BPDCN patients included CEBPB, CEBPD, CSF1R, MN1, SPI1, and RBPJ. In contrast, genes significantly expressed in the BPDCN patient samples included SPIB, TCF4, and IRF8, underlining their physiological activity in pDC (Supplementary Table S4, Figure 3D) [19]. Although BPDCN samples represent abnormal cells, they share many gene activities with their originating entity which may impact VENTX expression. Thus, this analysis confirmed and added factors and pathways to the list of potential VENTX regulators which finally contained the TFs CEBP, ID2, IRF2, IRF8, MN1, MYB, RYBP, SPI1, SPIB, and TCF4, and the CSF-, NOTCH-, and TNF/NFkB-signaling pathways.

### 2.3. Cell Line MUTZ-3 Serves as Model for VENTX-Positive cDC

Cell lines are useful in vitro models in which to analyze and modify gene expression, thereby uncovering gene regulatory networks. To find hematopoietic cell lines expressing VENTX, we screened RNA-seq dataset LL-100 which contains expression data for 100 leukemia/lymphoma cell lines [27]. This screening highlighted the myeloid cell line MUTZ-3 which expressed conspicuously high transcript levels of VENTX (Figure 1C). MUTZ-3 is derived from a patient with acute myelomonocytic leukemia [28], and serves as a suitable in vitro model for dendritic cell differentiation [29,30,31]. VENTX expression in MUTZ-3 cells was confirmed by RQ-PCR and Western blot analyses (Figure 1D,E). In comparison to primary cells, MUTZ-3 expressed similar VENTX transcript levels as monocytes and higher levels than progenitor-derived DCs while moDCs showed nigh absent VENTX expression (Figure 1D). Moreover, pDC-derived BPDCN cell line CAL-1 expressed VENTX just slightly (Figure 1D). Thus, for subsequent examinations, we used MUTZ-3 cells as a model.

**Figure 4:**
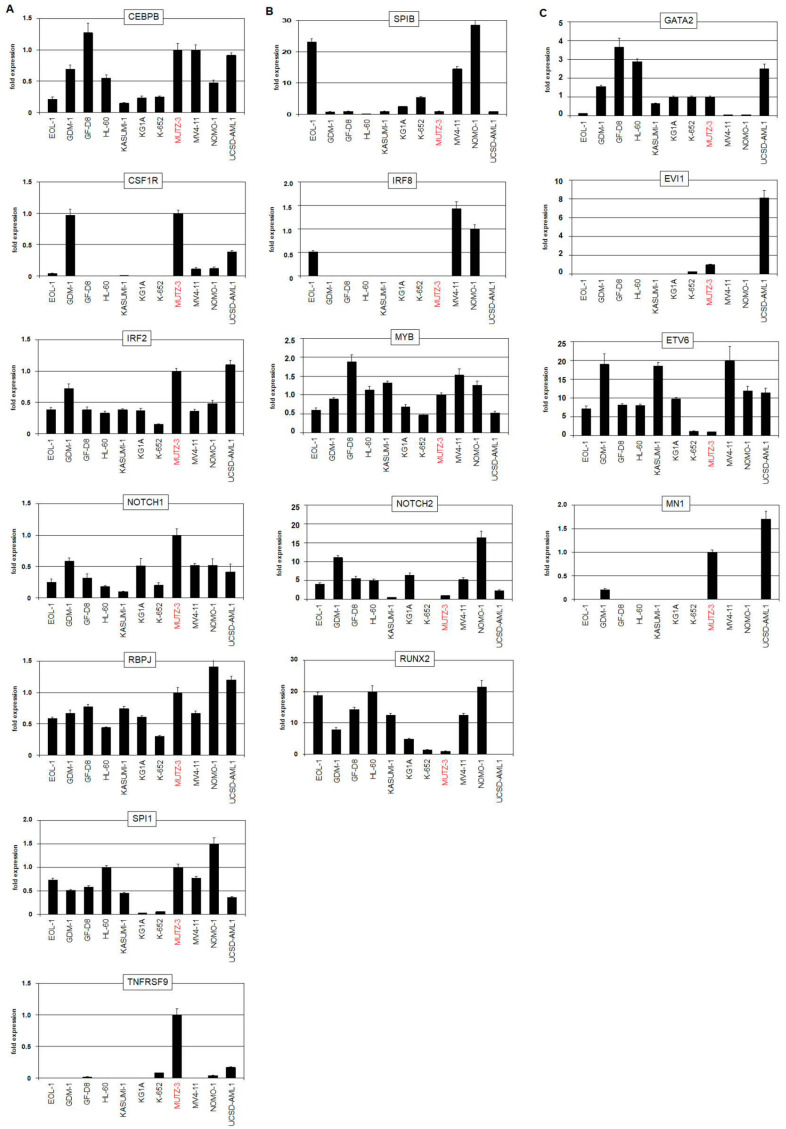
RQ-PCR analyses in MUTZ-3 and AML control cell lines. (**A**) RQ-PCR analyses of cDC-specific genes including CEBPB, CSF1R, IRF2, NOTCH1, RBPJ, SPI1, and TNFRSF9. (**B**) RQ-PCR analyses of pDC-specific genes including IRF8, SPIB, MYB, NOTCH2, and RUNX2. (**C**) RQ-PCR analyses of GATA2, EVI1, ETV6, and MN1 which are targeted by chromosomal aberrations in MUTZ-3 and UCSD-AML1. MUTZ-3 is highlighted in red.

Expression analysis of the above identified factors and pathways was performed using dataset LL-100 and by RQ-PCR of MUTZ-3 and selected AML cell lines. The data showed that MUTZ-3 expressed elevated CEBPA, CEBPB, CEBPD, CSF1R, CSF2RB, ID2, IRF2, NOTCH1, RBPJ, SPI1, and TNFRSF9 and reduced levels of IRF8, SPIB, and TCF4 while that of MYB was inconspicuous (Supplementary Table S5, Supplementary Figure S1A,B and Figure 4A,B). This gene signature realized in MUTZ-3 resembled that described for cDCs [32]. Moreover, extended analysis of reported genes discriminating two cDC subtypes revealed upregulated ESAM, ITGAM, ITGAX, and SIRPA and downregulated BATF3, BTLA, CD8A, CD86, FLT3, ITGAE, MYCL, NFIL3, NOTCH2, and RUNX2 (Supplementary Table S5, Supplementary Figure S1C and Figure 4B) [33]. Together, these data suggested that MUTZ-3 represents a cDC2-related cell line. LHCs have been proposed to derive from cDCs [24]. Therefore, data derived from LHC may help to understand the role of VENTX in DCs. Interestingly, published gene expression profiling data from LHCs showed significant VENTX activity as compared to normal Langerhans cells. However, additional DC-associated genes like CEBPB, CEBPD, CSF1R, IRF2, RBPJ, SPIB, SPI1, and TCF4 were not differentially expressed (Supplementary Table S6). Moreover, MUTZ-3 cells did not express the Langerhans cell marker CD207 (Supplementary Figure S2), excluding that MUTZ-3 represents cDC-derived LHCs.

MUTZ-3 cells show physiological and aberrant gene activities, the latter possibly deregulated by chromosomal abnormalities, which may influence the expression of VENTX as well. The gene encoding VENTX is located at chromosomal position 10q26. However, the previously described karyotype and genomic profiling data generated for this study from MUTZ-3 showed neither chromosomal nor genomic aberrations at the locus of VENTX (Supplementary Figure S3A) [34].

Nevertheless, MUTZ-3 contains two prominent rearrangements, namely inv(3)(q21q26) and t(12;22)(p13;q11), targeting GATA2 and EVI1, and ETV6 and MN1, respectively. Both aberrations deregulate their putative genes without generating gene fusions in this cell line [34,35,36]. RNA-seq expression data consistently showed reduced expression levels of GATA2 and ETV6 and elevated levels of EVI1 and MN1 for MUTZ-3 (Supplementary Figure S3B–D). AML cell line UCSD-AML1 also contains, in addition to t(12;22)(p13;q11), the inv(3)-synonymous rearrangement t(3;3)(q21q26) [37], expresses lower VENTX levels, but is not included in the LL-100 panel (Figure 1D). RQ-PCR analyses of MUTZ-3, UCSD-AML1, and controls demonstrated enhanced expression of EVI1 and MN1 in both cell lines while a transcriptional reduction of GATA2 and ETV6 was only detectable in MUTZ-3 (Figure 4C). GATA2 supports DC development, EVI1 is highly expressed in stem cells and downregulated in myelopoiesis, including DC differentiation, ETV6 acts as a pivotal factor in cDC1 differentiation, and MN1 operates as an oncogene in AML [32,38,39,40]. These four genes play basic roles in normal and aberrant myelopoiesis and may, therefore, impact VENTX expression levels in the normal and/or oncogenic context. Thus, we added GATA2, EVI, and ETV6 to the list of potential VENTX-regulators which already contained MN1.

### 2.4. Evaluation of VENTX Regulators

**Figure 5:**
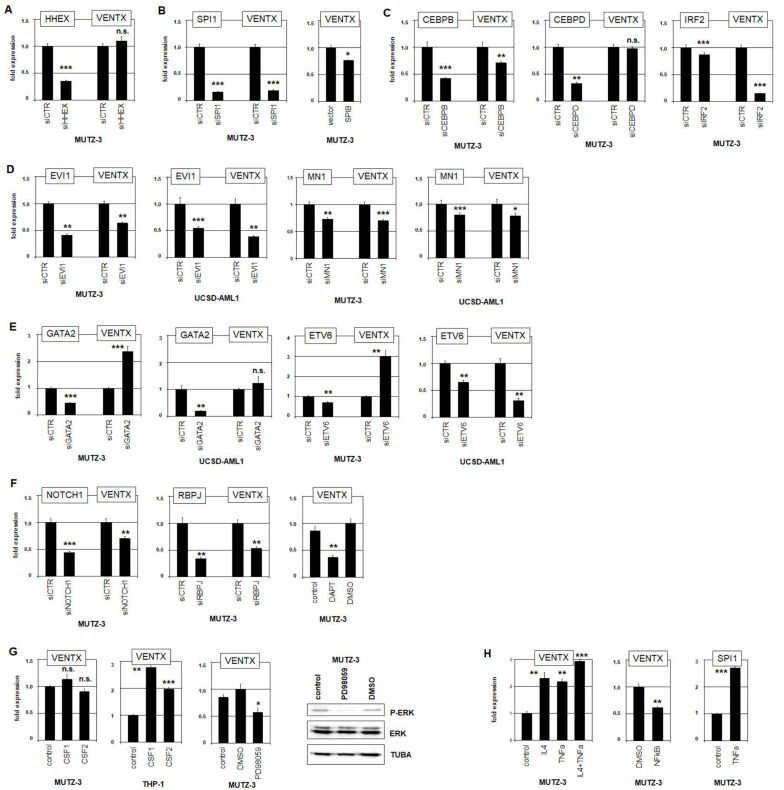
Evaluation of VENTX regulators. To evaluate identified candidate regulators of VENTX, we performed siRNA-mediated knockdown experiments and subsequent RQ-PCR analyses in MUTZ-3 and UCSD-AML1 cells. (**A**) Knockdown of HHEX showed no effect on VENTX levels in MUTZ-3. (**B**) Knockdown of SPI1 (left) and forced expression of SPIB (right) in MUTZ-3 resulted in reduced VENTX expression, indicating that SPI1 activated while SPIB repressed VENTX. (**C**) Knockdown of CEBPB and IRF2 resulted in reduced VENTX expression, indicating that both TFs activate VENTX in MUTZ-3. Knockdown of CEBPD (middle) showed no effect on VENTX expression. (**D**) Knockdown of EVI1 (left) and of MN1 (right) in MUTZ-3 and UCSD-AML1 resulted in reduced VENTX expression levels, indicating an activatory input of these factors in both cell lines. (**E**) Knockdown of GATA2 (left) and of ETV6 (right) in MUTZ-3 and UCSD-AML1 resulted in elevated VENTX expression levels in MUTZ-3 only. In UCSD-AML1, no effect or elevated VENTX levels were detected. (**F**) Knockdown of NOTCH (left) and of RBPJ (middle) resulted in reduced VENTX expression levels. Treatment of MUTZ-3 cells with NOTCH-inhibitor DAPT also resulted in reduced VENTX expression, supporting that NOTCH1-signaling activated VENTX expression in MUTZ-3. (**G**) Stimulation with CSF1 or CSF2 showed no significant effect on VENTX expression levels in MUTZ-3 cells (left) while THP-1 cells showed increasing VENTX levels (middle left). Treatment of MUTZ-3 cells with ERK-inhibitor PD98059 resulted in reduced VENTX expression levels (middle right). Western blot analysis showed reduced levels of phosphorylated ERK protein after PD98059 treatment. (**H**) Stimulation of MUTZ-3 cells with TNFa and/or IL4 resulted in elevated VENTX transcript levels (left). Treatment of MUTZ-3 with NFkB-inhibitor caused reduced VENTX expression (middle). Stimulation of MUTZ-3 with TNFa resulted in elevated expression levels of SPI1 (right). The calculated *p*-values are indicated by asterisks (* *p* < 0.05, ** *p* < 0.01, *** *p* < 0.001, n.s. not significant).

To evaluate all these identified potential regulators of VENTX expression, we performed siRNA-mediated knockdown experiments for selected TFs and stimulated cell lines with interleukins and inhibitors. Particular hematopoietic entities specifically coexpress NKL homeobox genes which regulate each other and participate in a gene network [6,7]. Therefore, we analyzed the potential impact of NKL homeobox gene HHEX which was detected in both pDC and cDC. However, siRNA-mediated knockdown of HHEX in MUTZ-3 showed no alteration of VENTX expression level, demonstrating that HHEX does not regulate VENTX (Figure 5A).

SPI1 and SPIB are members of the ETS family of TFs and important regulators of hematopoietic differentiation [41]. SiRNA-mediated knockdown of cDC-specific SPI1 in MUTZ-3 resulted in reduced VENTX expression levels, indicating that SPI1 operated as activator (Figure 5B). In contrast, forced expression of pDC-specific factor SPIB in SPIB-negative MUTZ-3 reduced VENTX expression, showing that SPIB acted as repressor (Figure 5B). Thus, these related TFs performed opposing activities in VENTX regulation. Moreover, public ChIP data for SPI1 from myeloid cell line HL-60 showed binding at the promoter region of VENTX, demonstrating a direct transcriptional impact (Supplementary Figure S4A). SiRNA-mediated knockdown of both CEBPB and IRF2 in MUTZ-3 resulted in reduced VENTX expression levels, indicating that these cDC-associated TFs also operated as activators (Figure 5C). Public ChIP data for CEBPB from myeloid cell line K-652 showed this factor to bind at the VENTX gene, indicating direct regulatory input (Supplementary Figure S4B). Interestingly, knockdown analysis of CEBPD demonstrated that this CEBPB-related factor did not regulate VENTX (Figure 5C). The role of IRF2 in differentiation of DCs has been reported before, while its potential impact in VENTX regulation is a novel finding [42,43].

EVI1, MN1, GATA2, and ETV6 are targeted by chromosomal aberrations in MUTZ-3 and UCSD-AML1. SiRNA-mediated knockdown of EVI1 or MN1 resulted in reduced VENTX expression in both cell lines, demonstrating an activatory impact for both factors (Figure 5D). In contrast, knockdown of GATA2 resulted in elevated VENTX expression in MUTZ-3 but showed no significant change in UCSD-AML1 (Figure 5E). Furthermore, siRNA-mediated knockdown of ETV6 caused VENTX elevation in MUTZ-3 but VENTX suppression in UCSD-AML1 (Figure 5E). ChIP-seq data for GATA2 from K-652 and for ETV6 from GM12878 cells indicated direct VENTX regulation by these TFs (Supplementary Figure S4C,D). Thus, knockdown experiments for GATA2 and ETV6 showed contrasting effects on VENTX activity in these cell lines which may underlie the lower VENTX transcript levels observed in UCSD-AML1.

To analyze the impact of the NOTCH-signaling pathway on VENTX expression, we performed siRNA-mediated knockdown of the receptor NOTCH1 and the associated TF RBPJ in MUTZ-3. Both experiments showed reduced levels of VENTX transcripts (Figure 5F). Consistently, treatment of MUTZ-3 cells with NOTCH-inhibitor DAPT mediated VENTX suppression as well, demonstrating that this pathway activates VENTX expression (Figure 5F). Interestingly, MUTZ-3 cells expressed high levels of NOTCH-ligand JAG1, indicating autocrine activation of this pathway (Supplementary Figure S1A). Reportedly, NOTCH-ligands JAG1 and JAG2 are expressed in DCs and play functional roles in immune responses [44,45,46].

Furthermore, MUTZ-3 cells require supernatant from 5637 cells for their growth in culture. This supernatant contains CSF1 (formerly G-CSF) and CSF2 (formerly GM-CSF) [47]. The cognate receptors CSF1R and CSF2RB are highly expressed in MUTZ-3 as shown by RNA-seq data and RQ-PCR analyses (Supplementary Figure S1A and Figure 4A). Stimulation of MUTZ-3 with additional recombinant CSF1 or CSF2 protein showed no effect on VENTX (Figure 5G). However, stimulation of VENTX-low and supernatant-independent AML cell line THP-1 with CSF1 or CSF2 resulted in increased VENTX levels, showing an activatory role (Figure 5G). Consistently, treatment of MUTZ-3 with ERK-inhibitor PD98059 resulted in reduced phosphorylation of ERK protein and downregulated VENTX expression (Figure 5G), indicating that CSF1/2-mediated ERK-signaling activated VENTX transcription.

CSF2, TNFa, and IL4 have been reported to induce dendritic cell differentiation in MUTZ-3 [28]. Here, additional stimulation of MUTZ-3 with TNFa and/or IL4 resulted in increased VENTX expression after already four hours (Figure 5H). Treatment of MUTZ-3 with NFkB-inhibitor mediated VENTX suppression, consistently showing that activatory TNFa-signaling operates via NFkB (Figure 5H). Moreover, TNFa-stimulation resulted in increased expression of SPI1 (Figure 5H), indicating that this TF may underlie the transcriptional activation of VENTX by this signaling pathway.

Taken together, these data showed that VENTX is activated by cDC-specific TFs CEBPB, IRF2, and SPI1. The DC-associated CSF-, NOTCH-, TNFa-, and IL4-signaling pathways mediated VENTX activation as well while the pDC-specific TF SPIB inhibited VENTX transcription. Furthermore, aberrantly overexpressed EVI1 and MN1 activated while aberrantly downregulated ETV6 and GATA2 mediated transcriptional repression of VENTX in MUTZ-3 cells. These data integrate VENTX in a network of regulators involved in DC differentiation processes.

### 2.5. Functional Analyses of VENTX in MUTZ-3 Cells

**Figure 6:**
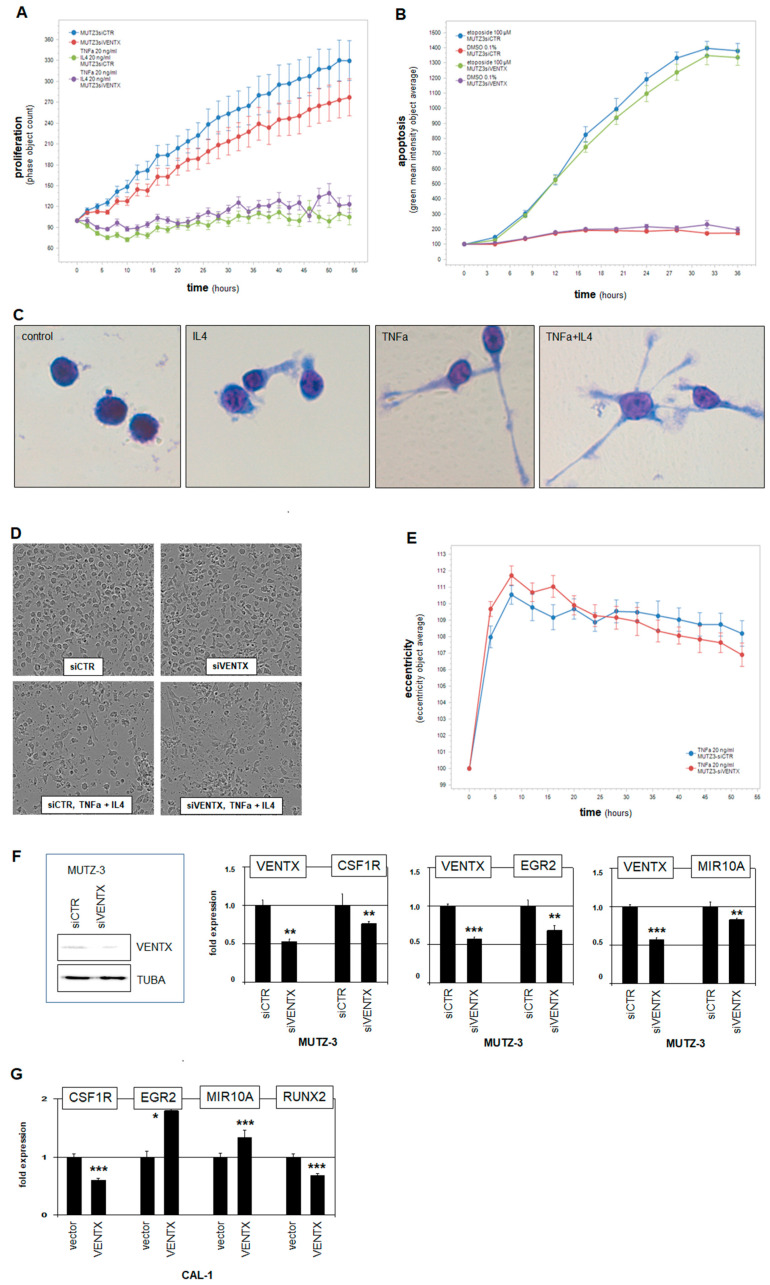
Functional analyses of VENTX. (**A**) MUTZ-3 cells treated for siRNA-mediated knockdown of VENTX were analyzed by live-cell imaging, demonstrating a supportive input for VENTX in proliferation. Additional stimulation with TNFa and IL4 inhibited proliferation but showed no difference between VENTX-knockdown and control. (**B**) Additional treatment with apoptosis-inducer etoposide increased apoptosis, showing no difference between VENTX-knockdown and control. (**C**) May–Grünwald–Giemsa staining of MUTZ-3 cells showed distinct morphological alterations after two days stimulation with IL4 and/or TNFa. These cell extensions and formations of dendrites were interpreted as dendritic cell differentiation. (**D**) Microscopic images of MUTZ-3 cells after VENTX-knockdown and stimulation with/without IL4 and TNFa performed by the live-cell imager. (**E**) TNFa-induced alteration of the cell morphology from MUTZ-3 was quantified using the eccentricity-tool from the live-cell imager. The eccentricity increased strongly after TNFa-stimulation. However, no difference between VENTX-knockdown and control cells were detected. (**F**) Western blot analysis of VENTX and TUBA-control confirmed reduced VENTX expression after knockdown at the protein level in MUTZ-3 cells (left). RQ-PCR analyses of CSFR1, EGR2, and MIR10A after VENTX-knockdown in MUTZ-3 cells demonstrated that VENTX activated these genes. (**G**) RQ-PCR analyses of CSFR1, EGR2, MIR10A, and RUNX2 after forced expression of VENTX in BPDCN cell line CAL-1. The calculated *p*-values are indicated by asterisks (* *p* < 0.05, ** *p* < 0.01, *** *p* < 0.001, n.s. not significant).

To study functional aspects of VENTX activity in DCs, we performed live-cell imaging of MUTZ-3 cells after siRNA-mediated knockdown of this homeobox gene. These experiments showed slightly reduced proliferation (Figure 6A), indicating that VENTX moderately promotes cell growth. To analyze the role of VENTX in survival processes, we additionally treated the knockdown cells with the apoptosis-inducer etoposide. However, these results showed no difference to the control (Figure 6B), indicating that VENTX is not involved in protection against apoptosis in MUTZ-3. To examine its role in differentiation processes, we stimulated the VENTX-knockdown cells with the reported differentiation-inducers TNFa and IL4. Generally, cell differentiation corresponds with reduced cell proliferation which was clearly detected after this treatment (Figure 6A). However, reduced VENTX in MUTZ-3 cells showed no significant difference in proliferation as compared to the control in this setting (Figure 6A). Stimulation with TNFa and/or IL4 effects distinct morphological alterations of MUTZ-3, including extension of the cells and formation of spikes and dendrites as shown here after 20 h by May–Grünwald–Giemsa staining of controls (Figure 6C) and by live-cell imaging of siRNA-treated cells (Figure 6D). However, quantification of TNFa-induced morphological alteration (eccentricity) showed no significant difference when VENTX expression was reduced (Figure 6E). Together, these experiments suggested that VENTX has no impact on phenotypical dendritic cell differentiation. Accordingly, comparison of public profiling data from DC-differentiated MUTZ-3 with our data from untreated cells showed that the expression level of VENTX was nearly unchanged (Supplementary Table S7), suggesting that VENTX may rather operate at DC-progenitor stages before terminal differentiation.

NKL homeobox gene VENTX encodes a TF which may, therefore, regulate DC-specific processes at the transcriptional level. The above identified differentially expressed genes in VENTX-positive cell line MUTZ-3 or primary cDC samples may regulate VENTX and/or represent VENTX target genes. Accordingly, RQ-PCR analysis of MUTZ-3 treated for siRNA-mediated knockdown of VENTX demonstrated CSF1R as activated target gene, implying mutual activation (Figure 6F). In contrast, HHEX, IRF8, MYB, NOTCH1, and SPI1 were not regulated by VENTX (Supplementary Figure S5A). To identify additional target genes, we performed gene expression profiling analysis of MUTZ-3 cells treated for siRNA-mediated knockdown of VENTX. Supplementary Table S8 shows the corresponding gene activities as log2-values and their calculated differences. Among the minimum twofold differentially expressed genes, we found several factors involved in dendritic cell differentiation and function and recognized, in addition, corresponding gene activities in primary cDCs or pDCs. CSF1R, EGR2, HIST1H3A, HOMER2, and NCOR2, which were activated by VENTX and elevated in cDCs (Supplementary Figure S5B), while HIST1H2BB, RUNX2, SMAD9, STAT3, and ZNF852 were inhibited by VENTX and more highly expressed in pDCs (Supplementary Figure S5C). RQ-PCR analyses of the selected genes EGR2 and MIR10A after siRNA-mediated knockdown confirmed their activation by VENTX in MUTZ-3 (Figure 6F). Forced expression of VENTX in pDC-derived BPDCN cell line CAL-1 resulted in altered expression levels of VENTX-target genes CSF1R, EGR2, MIR10A, and RUNX2 (Figure 6G). Except CSF1R, the analyzed genes were similarly regulated in both cDC and pDC cell line models, supporting the potential significance of VENTX in DC differentiation. Taken together, these results demonstrated a specific gene regulatory role for NKL homeobox gene VENTX in cDC differentiation and function. Our data support the hypothesis that VENTX operates mainly at DC-progenitor stages to drive differentiation of cDCs while suppressing pDC development.

## 3. Discussion

**Figure 7:**
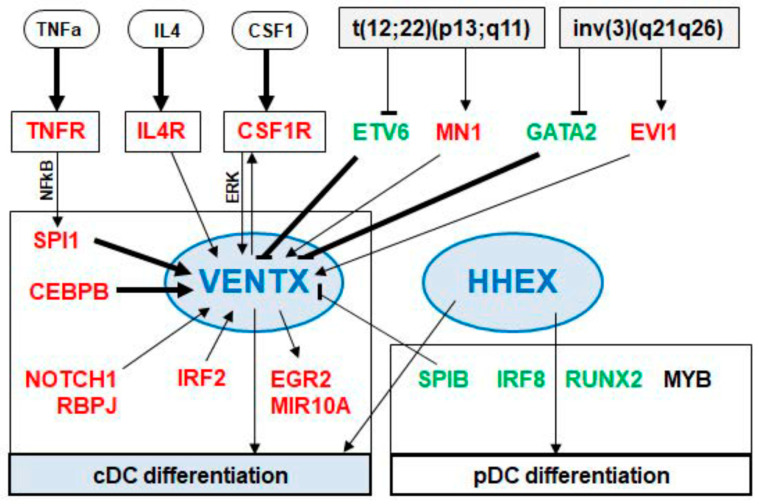
VENTX located in a gene regulatory network. This scheme summarizes the results of our study. The DC-associated NKL homeobox genes VENTX and HHEX are shown centrally involved in the differentiation of cDCs and pDCs. VENTX expression is activated by the TNFa-, IL4-, CSF1-, and NOTCH1-signaling pathways. The TFs SPI1, CEBPB, IRF2, MN1, and EVI1 activate VENTX while the TFs SPIB, ETV6, and GATA2 inhibit VENTX expression. Regulation by the TFs SPI1, CEBPB, ETV6, and GATA2 may operate directly as indicated by public ChIP-data (arrows in bold). The TFs ETV6, MN1, GATA2, and EVI1 are targeted by the chromosomal aberrations t(12;22)(p13;q11) and inv(3)(q21q26). Indicated VENTX target genes are CSFR1, EGR2, and MIR10A.

In this study, we analyzed the expression of NKL homeobox genes in progenitor-derived dendritic cells, showing that both cDCs and pDCs express HHEX while cDCs solely express VENTX. These screening results were included in our map, thus further extending the NKL-code for myelopoiesis. Accordingly, VENTX expression was detected in GMPs, granulocytes, monocytes, and cDCs while moDCs silenced VENTX during differentiation, expressing DLX2, HHEX, and HLX [7]. Thus, all types of DCs show a unique NKL homeobox gene signature. Due to the reported basic impacts of NKL homeobox genes in developmental processes, they may, therefore, regulate subtype-specific gene targets in progenitors and/or mature DCs. Here, we focused on the cDC-associated NKL homeobox gene VENTX. We identified MUTZ-3 which expresses higher levels of VENTX when compared to primary DCs but at levels similar to monocytes. This cell line represents an established model for dendritic cells [29,30,31], supporting our finding that VENTX is involved in differentiation and functional aspects of cDCs. Comparative gene expression analyses using datasets from normal and malignant DCs revealed several candidates which may regulate VENTX in DC development. Gene modulating experiments to evaluate these candidates as well as VENTX target gene analyses were performed in MUTZ-3 cells, generating an intricate network of TFs and signaling pathways as summarized in Figure 7. These results correspond to published studies which report specific factors and pathways for the development of cDCs and pDCs.

Our data indicated that SPI1 activated while SPIB repressed VENTX expression. SPI1 represents a master TF regulating myeloid development [48]. Moreover, SPI1 plays a central role in DC differentiation, performing gene regulation and protein interactions [22,49]. SPIB is a main factor for the development of pDCs [50]. Interestingly, additional factors identified as VENTX regulators are functionally connected with SPI1. GATA2 inhibits the transcription of SPI1, regulating myeloid differentiation [51,52,53,54]. Downregulation of SPI1 results in elevated expression of GATA2 in DCs, demonstrating an inhibitory effect of SPI1 on GATA2 [55]. EVI1 activates the transcription of SPI1 and interacts with SPI1 at the protein level [56,57]. SPI1 interacts with IRF2, coactivating their target genes [58]. NFkB binds the gene of SPI1 directly and activates its transcription [59]. The pDC-associated factor MYB represses the transcription of both SPI1 and CEBPB [60]. Thus, our data, together with results from the literature, support the view that SPI1 and the closely related SPIB represent key physiological factors for DC-differentiation including the regulation of VENTX expression.

TFs from the IRF-family represent additional basic players in the course of DC development. IRF2 regulates cDC development while IRF8 is involved in the differentiation of pDCs and subtype cDC1 [61,62,63]. Furthermore, IRF8 and SPIB are activated by pDC-specific factor TCF4 [64]. Therefore, our data showing elevated IRF2 expression in MUTZ-3 and activation of VENTX by IRF2 further supports the conclusion that both VENTX and this cell line are cDC-associated.

Furthermore, our data show that ETV6 operates as a repressor for VENTX in MUTZ-3 cells unlike the situation in UCSD-AML1. Of note, in contrast to MUTZ-3, the rearrangement t(12;22)(p13;q11) generates a fusion of the ETV6 and MN1 genes in UCSD-AML1 cells [36]. Therefore, ETV6 knockdown in this cell line may reduce both wild type ETV6 and the ETV6-MN1 fusion transcript which might have opposing effects on VENTX expression. However, ETV6 aberrations are frequent in BPDCN patients all of which silenced VENTX, indicating that ETV6 downregulation is not a major driver of VENTX activation [65]. Furthermore, this observed difference for ETV6 and MN1 in MUTZ-3 and UCSD-AML1 may apply to GATA2 and EVI1 as well. MN1 operated as activator in both cell lines while knockdown of GATA2 boosted VENTX levels in MUTZ-3 but not in UCSD-AML1. Therefore, the deregulated genes GATA2 and EVI1 may generate a fusion in UCSD-AML1 as well. However, GATA2 mutations cause DC deficiency, filling a developmental role for this factor in this context [66]. MN1 expression correlated with VENTX activity in our analysis of AML patients, supporting its VENTX activating potential. Mechanistically, MN1 may get recruited to particular TFs like SPI1 via p300 [40]. Moreover, CEBPB is a reported target gene of MN1 in myeloid transformation and is involved in cDC differentiation [67,68]. Thus, MN1 may activate VENTX transcription via the cDC-specific TFs SPI1 and CEBPB.

Our search for VENTX target genes revealed activation of CSFR1, a regulatory connection which has been described in DCs before [69]. Thus, VENTX and CSF-signaling represent mutual activators, highlighting their roles in cDC development. In addition, gene expression profiling revealed that EGR2, HOMER2, MIR10A, and NCOR2 were activated while RUNX2, SMAD9, STAT3, and ZNF852 were downregulated by VENTX. Most of these genes encode important factors operating in differentiation and function of DCs. EGR2 is induced in DC development and regulates immunogenicity therein [70]. MIR10A belongs to the miRNA-signature of DCs and regulates DC activation [71,72]. NCOR2 plays a central role in differentiation processes of both monocytes and DCs [73]. RUNX2 controls the differentiation of pDCs and is downregulated in cDCs [74]. STAT3 and STAT5 regulate IRF8 expression, thus controlling pDC differentiation [62]. The corresponding regulatory impact of VENTX on the selected target genes EGR2, MIR10A, and RUNX2 in pDC-derived cell line CAL-1 supports a role of this NKL homeobox gene in differentiation of cDCs.

Extended analysis of gene activities indicated that MUTZ-3 is related to the cDC2 subtype. Nevertheless, MUTZ-3 is morphologically unlike DCs. Therefore, our data may indicate that this acute myelomonocytic leukemia cell line is rather primed for differentiation towards a cDC2 cell but arrested at a progenitor stage. So far, no patients with cDC-derived malignancy have been described, while BDCPN is a rare pDC-derived neoplasm carrying a worse prognosis [23]. However, subsets of LHCs have been proposed to represent cDC-derived tumors [24,25,26]. However, additional analyses are needed to clarify whether these cases are related to cDC-precursors or, instead, derived from Langerhans cells which differ in their ontogeny. Our data may provide additional tools to better discriminate VENTX-positive AML from potential cDC-derived or -primed malignancies clinically. Like several other NKL homeobox genes, VENTX is aberrantly overexpressed in subsets of AML patients, revealing its oncogenic potential [7]. However, our functional analyses of VENTX in MUTZ-3 did not support an oncogenic role for this NKL homeobox gene, despite elevated expression levels. VENTX expression has been detected in embryonic stem and in progenitor cells, indicating a role in regulation of stemness [75,76]. This function may play a role in AML subsets which aberrantly express VENTX [7,77,78]. Therefore, VENTX may serve as a marker in possibly cDC-derived malignancies and does not appear to an oncogene in such cases. Studies reporting that DCs derive from CML and AML cells indicate distinct plasticity of malignant myeloid cells which may complicate the differentiation between AML and DC-derived malignancies [79,80]

Finally, the VENTX locus is absent in mice and rats, unlike humans, dogs, and chickens [81]. Thus, VENTX plays no obvious role in the differentiation of cDCs and monocytes in rodents. Two questions arise from these findings: what is the specific role of VENTX in human cDCs and other VENTX-positive myeloid entities? In addition, do rodents compensate the loss of VENTX in cDCs by another factor? However, VENTX has been analyzed in the embryogenesis of the frog *Xenopus*, showing that GATA activates VENTX which in turn regulates NOTCH1 expression [82,83]. Our data show that GATA2 inhibits and NOTCH1 activates VENTX expression in MUTZ-3 cells. Thus, the VENTX upstream regulator GATA is conserved while the relation to its target gene NOTCH1 differs between VENTX-positive vertebrates. Together, these data may indicate that the regulation and function of NKL homeobox gene VENTX is diverse in vertebrates and that its role in cDCs is a special feature, at least in humans.

## 4. Materials and Methods

### 4.1. Gene Expression Profiling and RNA Sequence Analyses

Public expression profiling datasets used in this study were generated by HG U133 Plus 2.0 gene chips from Affymetrix (High Wycombe, UK) and obtained from Gene Expression Omnibus (GEO, www.ncbi.nlm.nih.gov). We exploited dataset GSE24759 for analyses of developing and mature myeloid cells including dendritic cells setting a cut-off for positive/negative gene activity at 7.0 [84]. We used the online tool GEOR which extracts differentially expressed genes of two selected sample groups, generating volcano-plots for visualization and lists of significantly expressed genes for subsequent analysis. In addition, we analyzed RNA-seq based gene expression data from the Human Protein Atlas (www.proteinatlas.org) [85]. Dataset GSE89565 was analyzed for gene activities in BPDCN and AML patients and dataset GSE16395 was used to examine normal Langerhans cells and aberrant LHCs [24,86]. Recently, we sequenced transcriptomes from 100 leukemia/lymphoma cell lines including MUTZ-3 [27]. This LL-100 dataset is available from the European Nucleotide Archive (ENA; www.ebi.ac.uk/ena) via PRJEB30312. Comparative expression analysis of MUTZ-3 and 22 control cell lines was processed, analyzed, and normalized as described [27]. Genes were filtered for ≥50 normalized expression, average values for the control cell lines calculated, and the log2 fold changes to MUTZ-3 determined. For visualization of expression values, we used the public R-package shinyngs. Chromatin immuno-precipitation (ChIP)-sequencing (seq) data were obtained from GEO-datasets GSE32465 (CEBPB, GATA2, SPI1) and GSE96274 (ETV6) and analyzed using associated online tools and the Integrative Genomics Viewer (www.software.broadinstitute.org), respectively.

Expression profiling data from DC-differentiated MUTZ-3 cells were obtained from ArrayExpress (www.ebi.ac.uk/arrayexpress) via E-MEXP-3787 [29]. Expression profiling data from untreated and siRNA-treated MUTZ-3 cells were generated at the Genome Analytics Facility (Helmholtz Centre for Infection Research, Braunschweig, Germany). These expression profiling data were generated using HG U133 Plus 2.0 gene chips (Affymetrix) and are available from ArrayExpress via E-MTAB-10277. After RMA-background correction and quantile normalization of the spot intensities, the profiling data were expressed as ratios of sample means and subsequently log2 transformed. Data processing was performed via R/Bioconductor using public limma and affy packages.

### 4.2. Polymerase Chain-Reaction (PCR) Analysis

Total RNA was extracted from cultivated cell lines using TRIzol reagent (Invitrogen, Darmstadt, Germany). Primary human RNA was obtained commercially: we used total RNA of CD14-positive monocytes, dendritic cells derived from monocytes by stimulation with a mix of cytokines, and of directly isolated dendritic cells, all obtained from 3H Biomedical AB (Uppsala, Sweden). The derived cDNA was synthesized using 1 µg RNA, random priming, and Superscript II (Invitrogen), and applied for PCR analyses.

Real-time quantitative (RQ)-PCR analyses were performed using the 7500 Real-time System, and commercial buffer and primer sets (Applied Biosystems/Life Technologies, Darmstadt, Germany). For normalization of expression levels, we quantified the transcripts of TATA box binding protein (TBP). Quantitative analyses were performed at least twice and conducted in triplicate. The values obtained show representative experiments and indicated standard deviations as error bars. The statistical significance was assessed by Student’s *t*-test and the calculated *p*-values indicated by asterisks (* *p* < 0.05, ** *p* < 0.01, *** *p* < 0.001, n.s. not significant).

### 4.3. Western Blot Analysis

Western blots were generated by the semi-dry method. Protein lysates from cell lines were prepared using SIGMAFast protease inhibitor cocktail (Sigma, Taufkirchen, Germany). Proteins were transferred onto nitrocellulose membranes (Bio-Rad, München, Germany) and blocked with 5% dry milk powder dissolved in phosphate-buffered-saline buffer. The following antibodies were used: alpha-Tubulin (T6199, Sigma), VENTX (PA5-21006, Invitrogen, Darmstadt, Germany), ERK (sc-94, Santa Cruz Biotechnology, Heidelberg, Germany), and phospho(P)-ERK (sc-7383, Santa Cruz Biotechnology). For loading controls, blots were reversibly stained with Poinceau (Sigma) and detection of alpha-Tubulin (TUBA) performed thereafter. Secondary antibodies were linked to peroxidase for detection by Western-Lightning-ECL (Perkin Elmer, Waltham, MA, USA). Documentation employed the digital system ChemoStar Imager (INTAS, Göttingen, Germany).

### 4.4. Cell Lines and Treatments

Cell lines were retrieved from the stocks of the Leibniz-Institute DSMZ (Braunschweig, Germany) and cultivated as described elsewhere [87]. The BPDCN-derived cell line CAL-1 was kindly provided by Dr. Maeda from the Nagasaki University Graduate School of Biomedical Sciences and cultivated as reported [88]. To modulate gene expression levels, we used gene specific siRNA oligonucleotides and AllStars negative Control siRNA (siCTR) obtained from Qiagen (Hilden, Germany). Commercial gene expression constructs for SPIB and VENTX were cloned in vector pCMV6-XL5 and obtained from Origene (Wiesbaden, Germany). An empty vector served as control. SiRNAs (80 pmol) and expression constructs/vector controls (2 µg) were transfected into 1 × 10^6^ cells by electroporation using the EPI-2500 impulse generator (Fischer, Heidelberg, Germany) at 350 V for 10 ms. Electroporated cells were harvested after 20 h cultivation.

Additional modulations of gene activities were performed by treatments for 20 h with 10 µM *N*-[*N*-(3,5-Difluorophenacetyl)-*L*-alanyl]-*S*-phenylglycine t-butyl ester (DAPT) or 14 µM NFkB-inhibitor and for 4 h with 35 µM PD98059. These chemicals were obtained from Sigma and dissolved in DMSO. Cells were additionally treated with 20 ng/mL CSF1/M-CSF, CSF2/GM-CSF, TNFa, and IL4 for 20 h (R&D Systems, Wiesbaden, Germany). For cytological analyses, cells were stained with May-Grünwald-Giemsa as follows: cells were cultivated in glass chamber slides with cover (Lab-Tek, Rochester, NY, USA) and fixed for 5 min in methanol. Subsequently they were stained for 3 min with May-Grünwald’s eosine-methylene blue solution modified (Merck, Darmstadt, Germany) diluted in Titrisol (Merck), and for 15 min with Giemsa azure eosin methylene blue solution (Merck). Images were captured with an AXIO Scope A1 microscope using AxioCam MRc5 and software AxioVision 4.7 (Zeiss, Göttingen, Germany).

For functional studies, treated cells were analyzed using the IncuCyte S3 Live-Cell Analysis System (Essen Bioscience, Hertfordshire, UK). Apoptosis was induced by treatments with 100 µM etoposide (Sigma) and detected via the IncuCyte Caspase-3/7 Green Apoptosis Assay diluted at 1:2000 (Essen Bioscience). Elongated cell morphology was quantified using the eccentricity tool of the cell-by-cell software package (Essen Bioscience). Analyses were performed twice in duplicates taking four pictures per well and time point. The calculated standard deviations were indicated in the figures as bars.

### 4.5. Genomic Analyses

For genomic profiling of cell line MUTZ-3 genomic DNA was prepared by the Qiagen Gentra Puregene Kit (Qiagen). Labeling, hybridization, and scanning of Cytoscan HD arrays was performed at the Genome Analytics Facility, according to the manufacturer´s protocols (Affymetrix). Data were analyzed using the Chromosome Analysis Suite software version 3.1.0.15 (Affymetrix).

## Data Availability

The data presented in this study are openly available.

## Supplementary Materials

The following are available online at https://www.mdpi.com/article/10.3390/ijms22115902/s1, Figure S1: Specific gene activities for cDC, pDC and cDC2 using LL-100, Figure S2: Expression analyses in LCH and cell lines, Figure S3: Genomic profiling data from MUTZ-3 and RNA-seq data of LL-100, Figure S4: ChIP-seq data, Figure S5: VENTX target gene activities, Table S1: Comparative gene expression of cDC versus pDC (GSE24759), Table S2: Comparative gene expression of cDC versus CMP (GSE24759), Table S3: Comparative gene expression of monocytes versus CMP (GSE24759), Table S4: Comparative gene expression of BPDCN versus VENTX-positive AML (GSE89565), Table S5: Comparative gene expression of MUTZ-3 versus AML cell lines (PRJEB30312), Table S6: Comparative gene expression of LHC and LH (GSE16395), Table S7: gene expression analysis in MUTZ-3 (E-MEXP-3787), Table S8: Expression profiling data of MUTZ-3 cells treated for siRNA-mediated VENTX knockdown (E-MTAB-10277).
