# Supplementary figures and images for: NKL Homeobox Gene VENTX Is Part of a Regulatory Network in Human Conventional Dendritic Cells
Source: Int J Mol Sci. 2021 May 31;22(11):5902. doi: 10.3390/ijms22115902 (PMC8198381; doi:10.3390/ijms22115902)

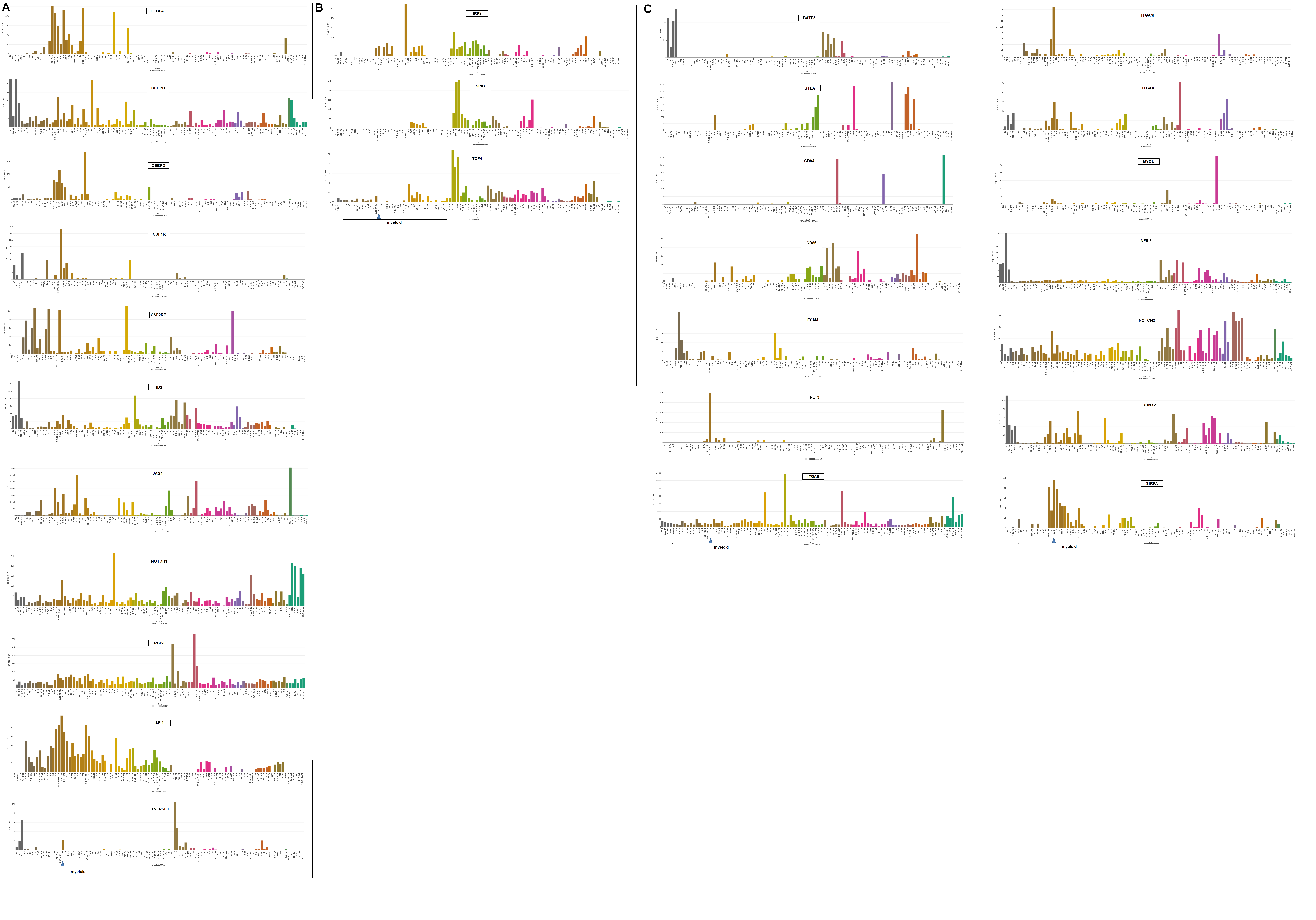

Supplement: Supplementary file 1 [file ijms-22-05902-s001.zip › SupplFig1_LL100 data.tif]

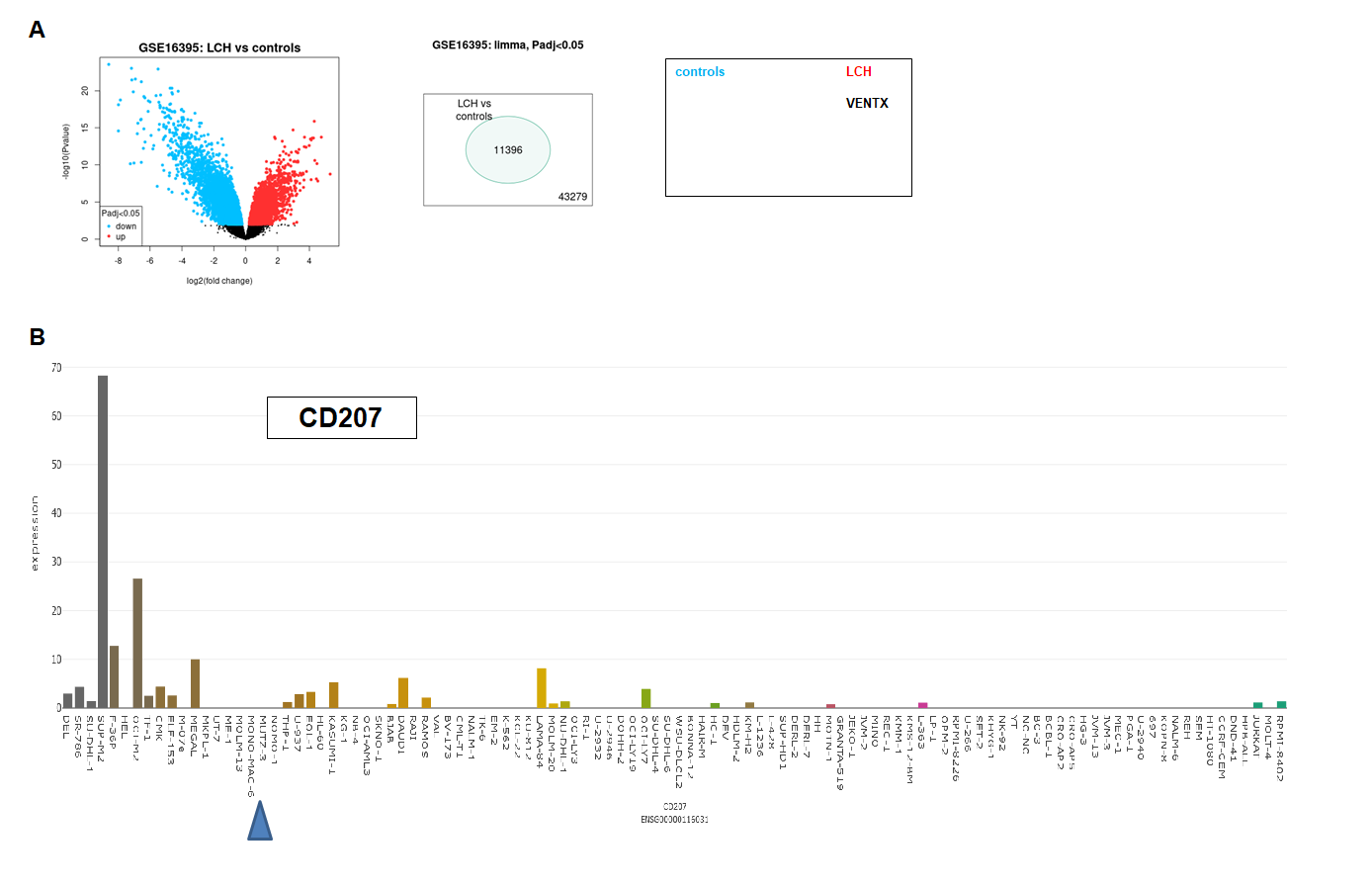

Supplement: Supplementary file 1 [file ijms-22-05902-s001.zip › SupplFig2_comparison LHC GSE16395.tif]

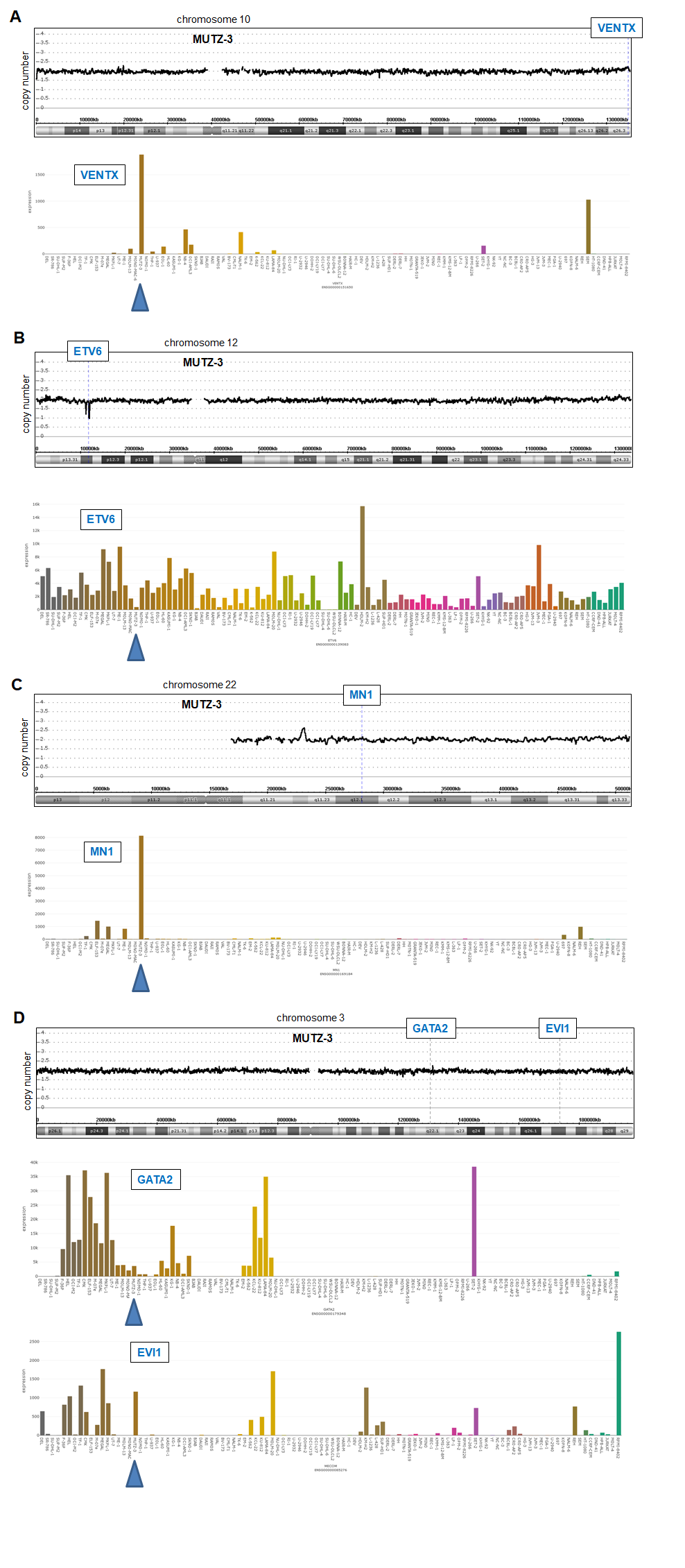

Supplement: Supplementary file 1 [file ijms-22-05902-s001.zip › SupplFig3_genomic profiling data.tif]

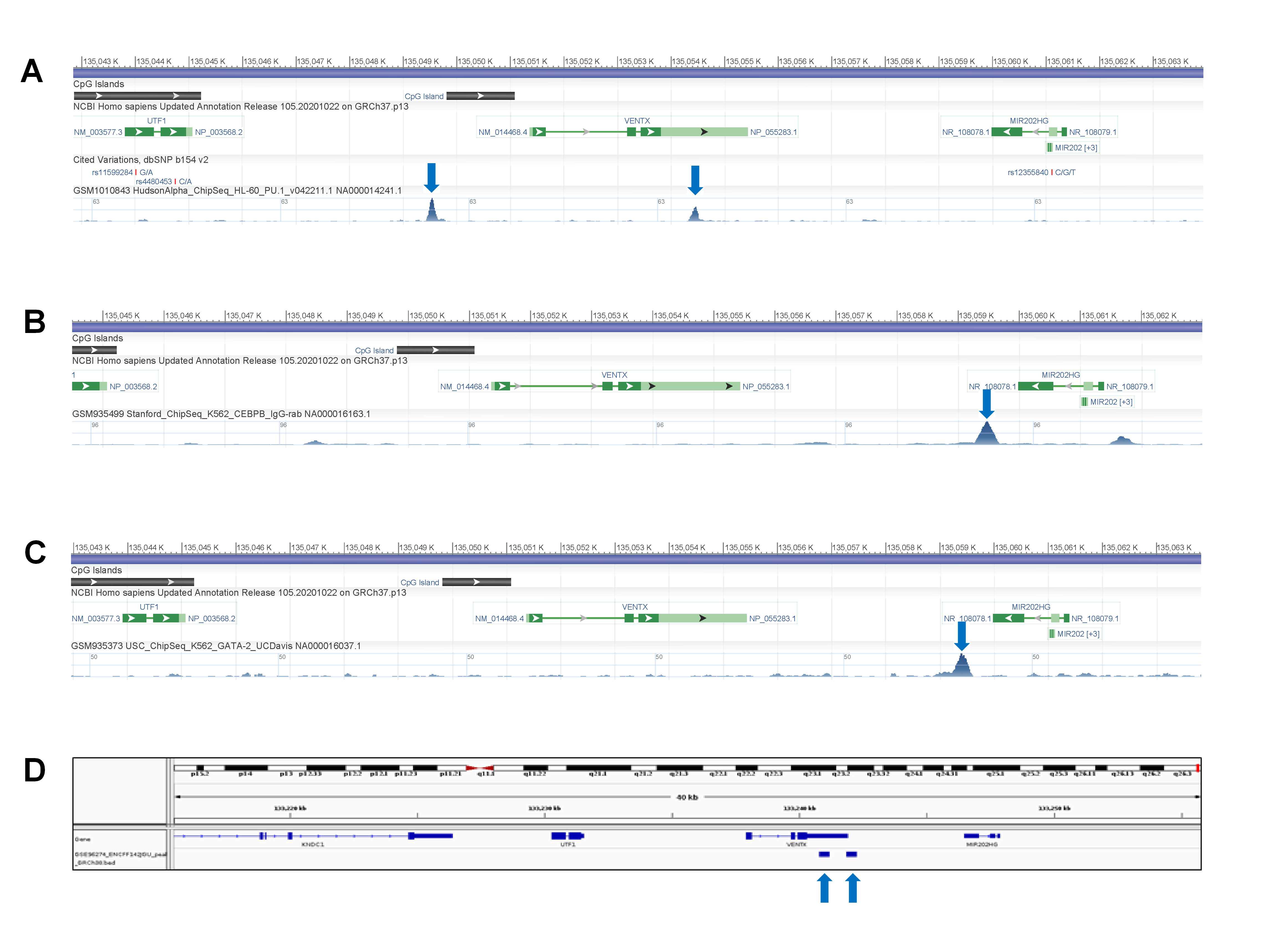

Supplement: Supplementary file 1 [file ijms-22-05902-s001.zip › SupplFig4_ChIP-seq data.tif]

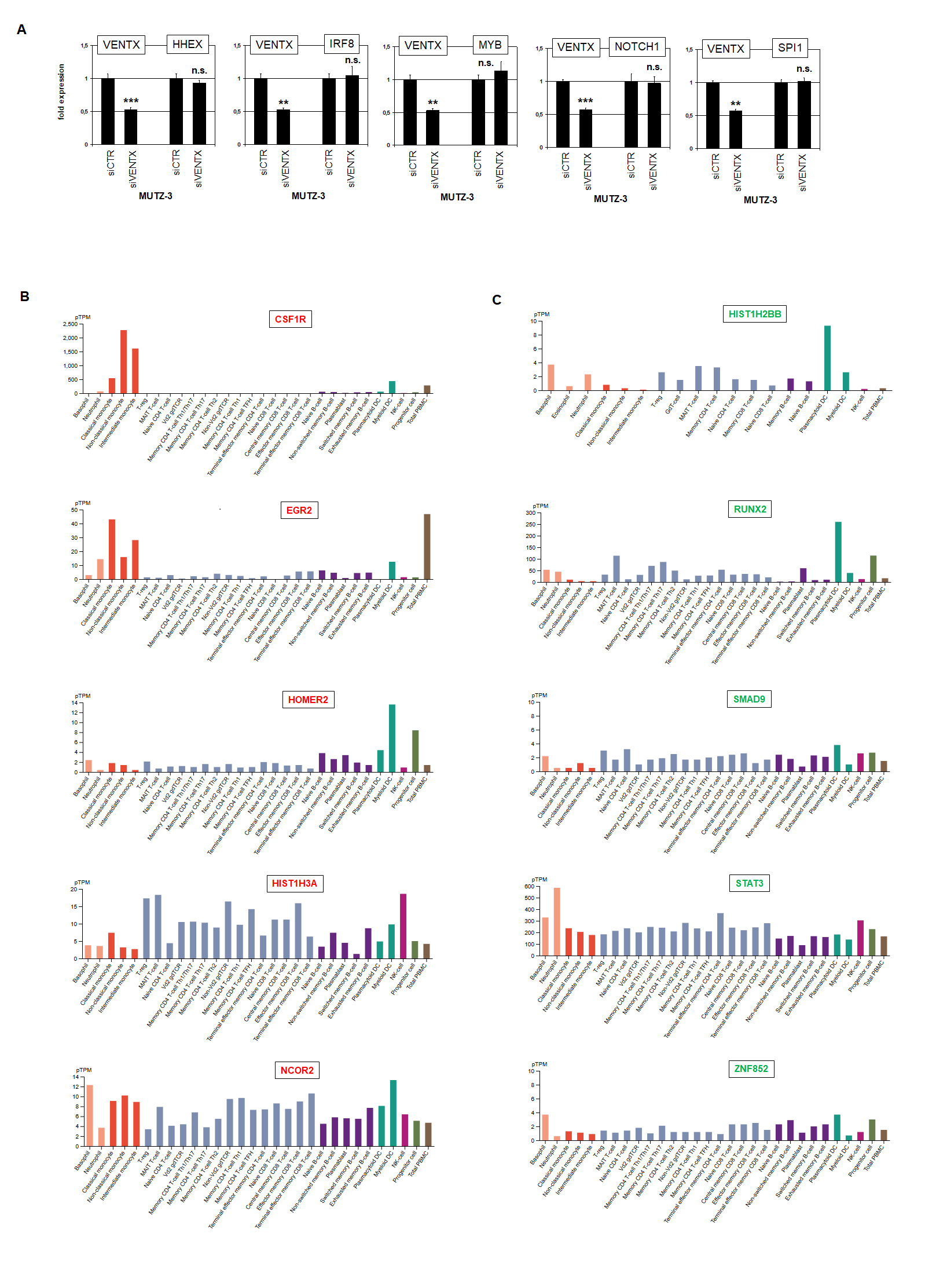

Supplement: Supplementary file 1 [file ijms-22-05902-s001.zip › SupplFig5_VENTX target gene activities in DCs.tif]
